# Effect of age on the pathogenesis of duck tembusu virus in Cherry Valley ducks

**DOI:** 10.3389/fmicb.2015.00581

**Published:** 2015-06-08

**Authors:** Ning Li, Chuanwei Lv, Ruichao Yue, Ying Shi, Liangmeng Wei, Tongjie Chai, Sidang Liu

**Affiliations:** College of Animal Science and Veterinary Medicine, Shan Dong Agricultural UniversityTai’an, China

**Keywords:** duck tembusu virus, Cherry Valley ducks, age, pathogenesis, immune response

## Abstract

The effect of host age on the outcome of duck tembusu virus (DTMUV) infection was studied in ducks. Three groups of Cherry Valley ducks at 1, 3, and 7 weeks of age were intramuscularly infected with DTMUV to systematically observe the clinical symptoms, pathological changes, tissue viral loads, and immune responses. Severe clinical symptoms and neurological dysfunction were observed in 1-week-old ducks as early as 2 days post infection (dpi) and some died at 5–7 dpi. Three weeks-old ducks showed similar but milder symptoms and no deaths. However, 7-weeks-old ducks showed only transient loss of appetite. Gross lesions gradually reduced in severity as ducks matured. One-week-old ducks showed endocardial hemorrhage, splenomegaly, swelling in the lymph follicles of the ileum, liver, and kidney swelling with degeneration, and meningeal hyperemia. Three-weeks-old ducks showed only mild pathological lesions. No visible lesions were observed in 7-weeks-old ducks. However, pathological histology analysis demonstrated all infected ducks displayed viral encephalitis. DTMUV could be detected in the brains of 1-week-old ducks as early as 1 dpi and virus titers of most organs in 1-week-old ducks were significantly higher than that of 3- and 7-weeks-old ducks at 3–5 dpi. The patterns of IFN-γ, IL-2, and serum neutralizing antibodies were similar, and there were significant difference between the youngest ducks and the older ducks at early infection stage (*P* < 0.05). More important is that although the antibody titers of all infected ducks were similar from 9 to 17 dpi, reduced clearance of virus was observed in the youngest groups comparing with the other two groups, indicating that immune system maturity was more important than the presence of neutralizing antibody. In summary, this study demonstrates that viral pathogenesis is strongest in 1-week-old ducks and the age-related immune response plays an important role in the pathogenesis of DTMUV in ducks.

## Introduction

Duck tembusu virus (DTMUV) can result in an acute, contagious infection characterized by decline in egg production of laying ducks (Su et al., 2011). The infectious disease was first detected in Shanghai in April 2010, and it spread widely throughout the main duck-rearing region in China (Yan et al., 2011). DTMUV belongs to the Ntaya virus group within the family Flaviviridae and possesses a positive-sense single-stranded RNA genome of approximately 11 kb in length (Kuno et al., 1998; Tang et al., 2012). Currently, almost all species of ducks are vulnerable to infection with DTMUV, especially Shelducks, which are most susceptible to this virus (Yan et al., 2011; Li et al., 2012; Tang et al., 2015). Moreover, chickens and geese could also be infected with this virus (Liu et al., 2012; Yun et al., 2012; Huang et al., 2013; Chen et al., 2014). Infected commercial ducks demonstrated loss of appetite, retarded growth, and neurological symptoms, while laying ducks displayed a severe drop in egg production. Autopsies showed ovarian hemorrhage and necrosis, endocardium and epicardium hemorrhage, liver degeneration, and swelling, and spleen necrosis (Cao et al., 2011; Su et al., 2011; Yan et al., 2011).

Although DTMUV was first isolated from egg ducks, differently aged ducks could be infected with the virus, especially ducks less than 3 weeks-old (Yun et al., 2012). We noted from the large numbers of clinical cases in which the morbidity and mortality of younger ducks were significantly higher than the older, that DTMUV obviously had differing pathogenicity for differently aged ducks. Moreover, the descriptions of necropsy and pathological changes of this disease were ambiguous. Therefore, the objective of this study was to investigate the effect of age on the pathogenesis of DTMUV in three different ages of Cherry Valley ducks to verify histopathological changes, virus distribution, and host immune response.

## Materials and Methods

### Virus

The FX2010 strain of DTMUV was used in this study, which was isolated from the clinical infected ducks in Fengxian district, Shanghai city in 2010 (Yan et al., 2011) and donated by Zejun Li researcher from Shanghai Veterinary Research Institute, the Chinese Academy of Agricultural Sciences. Virus stocks were propagated in SPF embryonated chicken eggs as previously described (Yan et al., 2011). Virus titer was determined as 10^6.1^ median tissue culture infective dose (TCID_50_)/mL by infection of duck embryo fibroblasts and calculation of the titers by the Reed and Muench method (Reed and Muench, 1938).

### Animals

One-day-old Cherry Valley ducks were obtained from Liu He Duck Farm (Shandong, China). The ducks were housed in SPF chicken isolators that were ventilated under negative pressure. Feed and water were provided ad libitum. Samples were collected from ducks before inoculation to ensure that the ducks were serologically and virologically DTMUV-negative as determined by a blocking ELISA and RT-PCR (Su et al., 2011; Li et al., 2012), respectively. Care and maintenance of all ducks were according to the guidelines of the Committee on the Ethics of Animal of Shandong and the biosecurity guidelines. All possible measures were taken to minimize differences in experimental conditions.

### Pathogenicity of DTMUV in Ducks

Eighty-one-days-old ducks were randomly divided into two groups of 50, and housed in the isolators separately. The ducks in group 1 were used for an infection experiment at 1 week, the other one was control group. Forty ducks in group 1 were intramuscular injection with 0.4 ml of virus stocks, control group were inoculated with 0.4 ml sterile phosphate-buffered saline (PBS) in the same manner. Ducks were observed continuously for 21 days, clinical symptoms and results of the autopsy were recorded. On 1, 3, 5, 7, 9, 11, 13, 15, 17, 19, and 21 day post-inoculation (dpi), three ducks from each group were euthanized and necropsied. Serums were collected and stored at -20^∘^C. Parts of the tissues (heart, liver, spleen, lung, kidney, and brain) were fixed with 10% neutral buffered formalin solution for histopathological examination, the rest were stored at -80^∘^C for RNA extraction. The remaining ducks at the end of this study were euthanized by the intravenous administration of sodium pentobarbital (100 mg/kg body weight).

One-day-old ducks were raised in isolators until 3- and 7-weeks-old, respectively. The experimental design was the same as that of 1-week-old ducks.

### RNA Extraction and Quantitative Real-Time RT-PCR

Total RNA was extracted from the collected tissues using Trizol reagent (Invitrogen, Carlsbad, CA, USA) following the manufacturer’s instructions. The concentration of each sample was measured using an ultraviolet spectrophotometer (Shimadzu, Shimazu, Japan). First cDNA was synthesized from 1 μg total RNA using PrimeScript ^TM^ RT Regent Kit with gDNA Eraser Kit (TaKaRa, Dalian, China). The primers for viral *E* gene were designed as previously reported (Yu et al., 2012). For confirming the viral copy numbers in the infected ducks, the viral titers (log10) were normalized to 1 μg of total RNA (Sun et al., 2014). Quantitative RT-PCR (qRT-PCR) was performed with the Applied Biosystems 7500 Fast Real-Time PCR System (Applied Biosystems, CA, USA) using the SYBR Green PCR kit (Takara, Dalian, China). qRT-PCR was conducted in a total volume of 20 μl following the manufacturer’s instructions. PCR reaction conditions consisted of 95^∘^C for 30 s, 40 cycles of amplification at 95^∘^C for 5 s, and 60^∘^C for 34 s, followed by a dissociation curve analysis step. Each sample was analyzed in triplicate.

### Detection of IFN-γ, IL-2, and Serum Neutralizing Antibodies

The serums were detected by using the ELISA Kit of duck IL-2 and IFN-γ (Langton, Shanghai, China) following the manufacturer’s instruction. The serum neutralizing antibodies were determined by a blocking ELISA (Li et al., 2012).

### Statistical Analysis

All data were expressed as means ± SD and processed by GraphPad Prism 5.0 (GraphPad Software Inc., San Diego, CA, USA). One-way ANOVA with Duncan’s multiple range test was used for evaluating data using SAS 9.1 software (SAS Institute, Inc., Cary, NC, USA). Statistical significance was set at *P* < 0.05.

## Results

### Clinical Signs and Gross Lesions

No clinical symptoms or necrotic lesions were observed throughout the experiment in control ducks. There were about twenty infected ducks showed loss of appetite and depression in the youngest challenged group after 2 dpi. At 3–4 dpi, almost 80% of ducks were reluctant to move, and showed white water-like diarrhea. At 5 dpi, six infected ducks died and three more were also dead at 7 dpi, moreover, about five infected ducks developed neurological signs, characterized by dystaxia and paralysis. At 9 dpi, the symptoms of the infected ducks gradually disappeared. In 3-weeks-old ducks inoculated with DTMUV, inappetence and decreased activity were observed from about eighteen ducks at 3 dpi. At 6 dpi, five ducks showed mild neurological signs, characterized by walking instability. At 7 dpi, feed intake in this group gradually recovered. Furthermore, no mortality was observed during the experiment. In 7-weeks-old ducks, about 20% infected ducks showed transient loss of appetite and depression at 3 dpi, but soon recovered at 6 dpi. No ducks displayed neurological symptoms or succumbed to infection.

**FIGURE 1:**
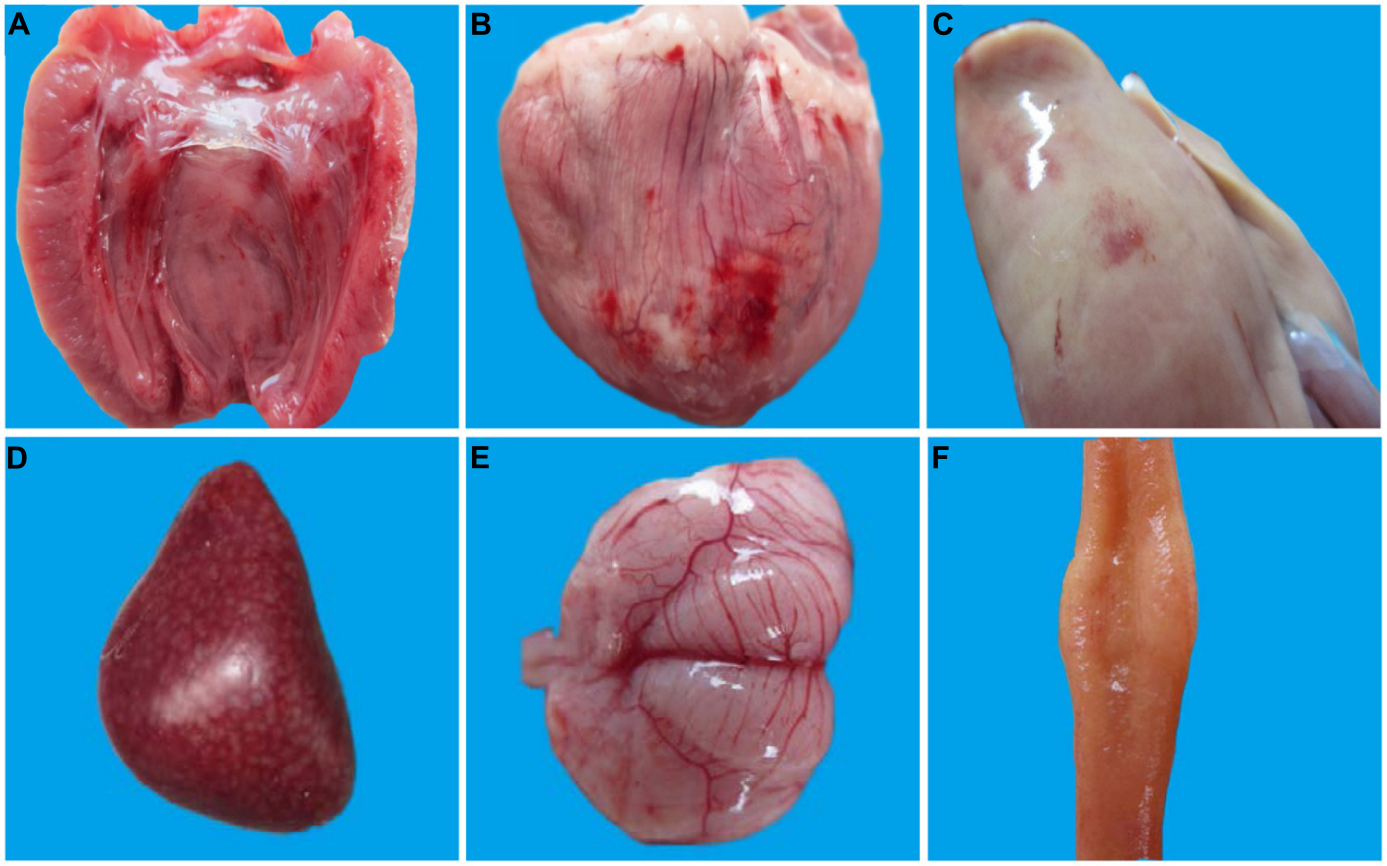
**Pathological changes of 1-week-old ducks infected with DTMUV**. The multiple tissues from 1-week-old ducks were detected at 5 dpi. **(A)** Endocardium showing hemorrhage; **(B)** Epicardium showing hemorrhage; **(C)** Liver is enlarged with blood spots; **(D)** Spleen is enlarged with mottled surface; **(E)** Meninx is congestive; **(F)** Mucosal swelling of the lymphoid follicles of the ileum.

At necropsy, bleeding of endocardium and epicardium of 1-week-old infected ducks was a significant lesion (**Figures 1A,B**) and they also demonstrated a swollen liver with hemorrhage dot (**Figure 1C**), splenomegaly with mottled surface (**Figure 1D**), meningeal hyperemia (**Figure 1E**), and mucosal swelling of the lymphoid follicles of the ileum (**Figure 1F**). Taken together, gross lesions were similar between the 1- and 3-weeks-old ducks, but the latter displayed milder lesions with a lower lesion detection rate. No visible lesions were found in control ducks and 7-weeks-old ducks infected with DTMUV.

### Pathological Histology Analysis

**FIGURE 2:**
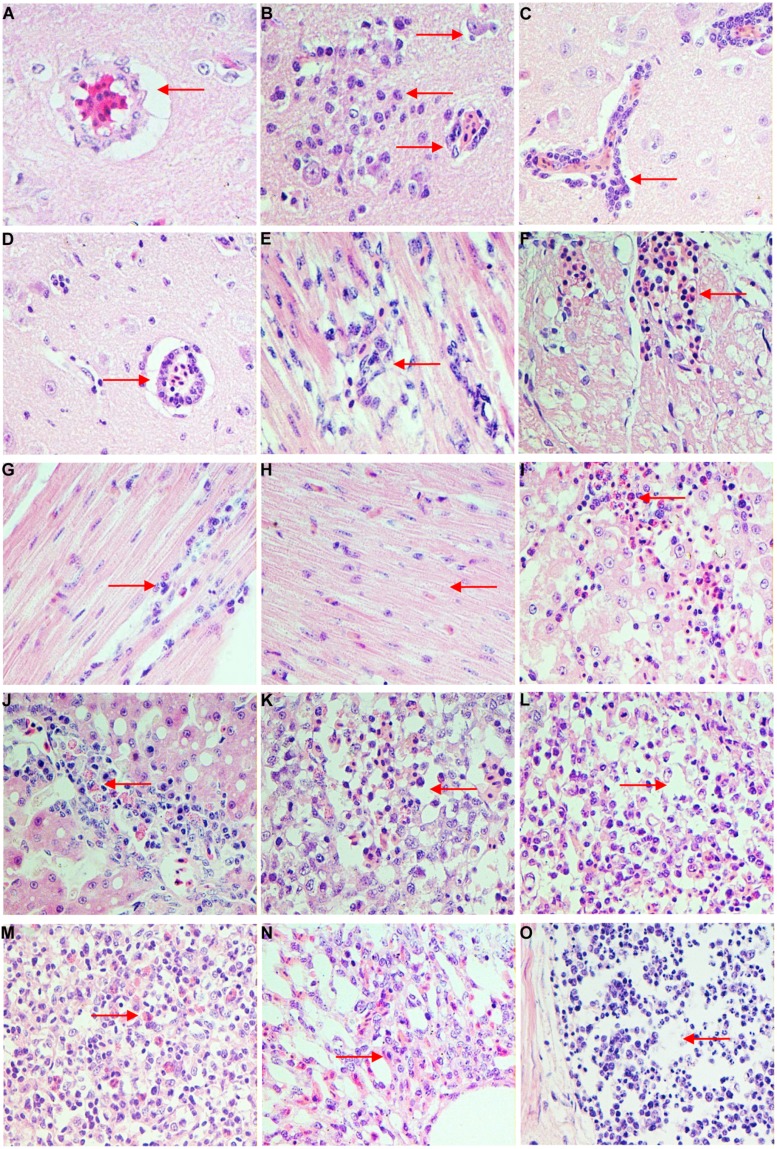
**Histopathologic changes of different age ducks infected with DTMUV. (A)** 1-week-old ducks, brain, 3 dpi: cerebral vascular endothelial cells swelling; **(B)** 1-week-old ducks, brain, 5 dpi: typical viral encephalitis; **(C)** 3-weeks-old ducks, brain, 7 dpi: severe perivascular inflammatory infiltrates; **(D)** 7-weeks-old ducks, brain, 7 dpi: mild perivascular inflammatory infiltrates; **(E)** 1-week-old ducks, heart, 3 dpi: degeneration and necrosis of myocardial fibers; **(F)** 1-week-old ducks, heart, 5 dpi: necrosis and bleeding of myocardial fibers; **(G)** 3-weeks-old ducks, heart, 5 dpi: granular degeneration and mild lymphocytic infiltration; **(H)** 7-weeks-old ducks, heart, 5 dpi: degeneration of myocardial fibers; **(I)** 1-week-old duck, liver, 5 dpi: severe degeneration and necrosis of liver, with infiltrating by heterophilic granulocyte; **(J)** 3-weeks-old ducks, liver, 5 dpi: degeneration and some inflammatory cells infiltration; **(K)** 1-week-old ducks, spleen, 5 dpi: lymphocytes of white pulp significant reduction and interstitial bleeding; **(L)** 3-weeks-old ducks, spleen, 5 dpi: lymphocytes of white pulp reduced; **(M)** 7-weeks-old ducks, spleen, 5 dpi: heterophilic granulocyte infiltration in white pulp; **(N)** 1-week-old ducks, lung, 7 dpi: interstitial pneumonia; **(O)** 1-week-old ducks, ileum, 5 dpi: necrosis of lymphocytes in lymphoid follicle.

Histological analysis showed that the differently aged ducks inoculated with DTMUV had different histopathological changes. In 1-week-old ducks, cerebral vascular hyperemia and endothelial cell degeneration and swelling were found in the brain at 3 dpi (**Figure 2A**). Perivascular inflammatory infiltrates, glial nodules, and neuronophagia were observed at 5 dpi in the dead ducks (**Figure 2B**), while at this time, the 3-weeks-old ducks showed widened gaps around small blood vessels. At 7 dpi, viral encephalitis became more severe in the youngest group, while the 3- and 7-weeks-old ducks demonstrated obvious but milder encephalitic lesions (**Figures 2C,D**). At 9 dpi, the viral encephalitis in the ducks of all three ages gradually decreased.

In the heart, 1-week-old ducks showed granular degeneration of myocardial fibers and lymphocytic infiltration at 3 dpi (**Figure 2E**). However, the older inoculated groups showed no significant lesions. At 5 dpi, degeneration and necrosis with scattered bleeding were observed in the youngest group of ducks (**Figure 2F**), 3 weeks-old ducks showed granular degeneration and mild lymphocytic infiltration (**Figure 2G**), but only cellular degeneration could be observed in the oldest ducks (**Figure 2H**). Myocardium hemorrhage and a small amount of inflammatory infiltration were observed in 1-week-old ducks, but ducks in the other two groups merely showed granular degeneration at 7–9 dpi. No significant histological changes were observed in any infected ducks at 11 dpi.

In the liver, 1-week-old ducks displayed vacuolated degeneration of liver cells at 3 dpi, followed by granular degeneration in 3-weeks-old ducks, but 7-weeks-old ducks had no obvious lesions. At 5–7 dpi, examinations of the youngest ducks revealed severe vacuolated degeneration, necrosis, scattered bleeding and heterophilic granulocyte infiltration (**Figure 2I**), while ducks in the other groups displayed moderate vacuolated degeneration, and heterophilic granulocyte and lymphocyte infiltration (**Figure 2J**). At 9–11 dpi, degeneration of the liver decreased but the inflammatory infiltration increased in all infected ducks.

In the spleen, obvious lymphocytic necrosis was observed in 1-week-old ducks infected with DTMUV at 3 dpi but not in the other experimental groups. The number of lymphocytes in the white pulp decreased significantly with bleeding and heterophilic granulocyte infiltration in the youngest group of ducks at 5 dpi (**Figure 2K**). This was also observed in the 3-weeks-old ducks, with decreased lymphocytes (**Figure 2L**), but the eldest group of ducks demonstrated heterophilic granulocyte infiltration (**Figure 2M**). At 7 dpi, all groups inoculated with DTMUV showed plentiful heterophilic granulocyte infiltration.

In the lung, blood capillary congestion of pulmonary alveoli was observed in 1-week-old ducks at 1–3 dpi. The youngest infected ducks displayed interstitial pneumonia with excessive inflammatory cell infiltration (**Figure 2N**), while the older groups only showed blood capillary congestion of pulmonary alveoli at 5–9 dpi.

In the kidney, at 5–9 dpi, vesicular degeneration was observed in the epithelium cells of the renal tubules. Moreover, necrosis, and interstitial hemorrhage were detected in the youngest group of ducks. In contrast, 3- and 7-weeks-old ducks showed only granular degeneration.

Finally, inflammatory cell infiltration and mild necrosis of the lymphocytes were observed in the lymphoid follicles in the mucosal lamina propria of the ileum at 3 dpi in all three age groups. However, at 5–7 dpi, significant necrosis of lymphocytes was observed in 1-week-old ducks (**Figure 2O**), while the older inoculated ducks showed only lymphoid hyperplasia and mild necrosis.

In summary, no microscopic lesions were observed in any control group, but the most severe lesions were observed in 1-week-old ducks, followed by those 3-weeks-old ducks, and finally 7-weeks-old ducks displayed mildest lesions, but differently aged infected ducks all showed viral encephalitis.

### Replication of DTMUV in Ducks

**FIGURE 3:**
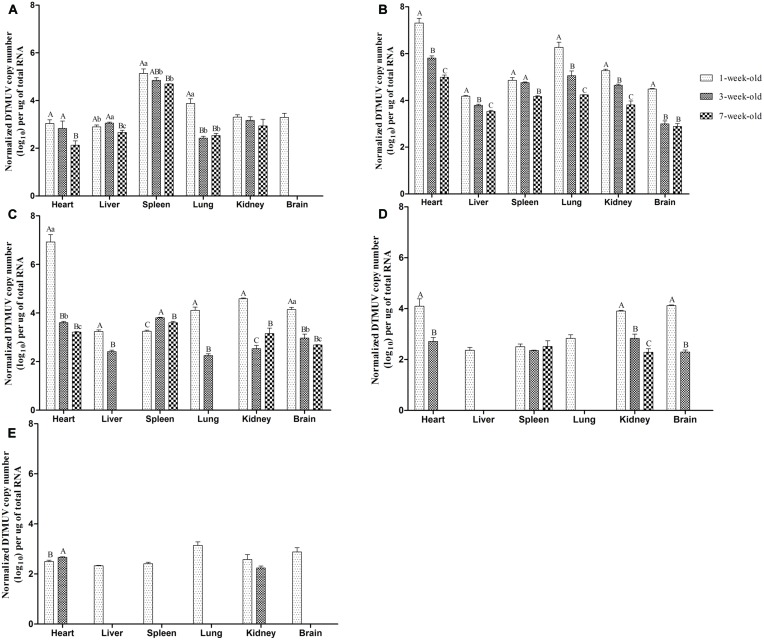
**Viral titers in tissues of different age ducks infected with DTMUV. (A–E)** Represent viral titers of multiple tissues from differently aged ducks infected with DTMUV at 1, 3, 5, 7, and 9 dpi, respectively. Data are expressed as mean ± SD (*n* = 3). Each sample was analyzed in triplicate. In each panel, different capital letters mean highly significant difference (*P* < 0.01), and different small letters mean significant difference (*P* < 0.05), while no letters mean no significant difference (*P* > 0.05). Significant differences were calculated by ANOVA with Duncan’s multiple range test.

The results of qRT-PCR to detect DTMUV copy numbers in tissues were shown in **Figure 3**. No viral RNA was detected in any control ducks. As shown in **Figure 3A**, DTMUV could be detected in all tested tissues (heart, liver, spleen, lung, kidney, brain) in 1-week-old ducks at 1 dpi. Of particular note was that the concentration of virus reached 10^3.3^ copies in the brain, yet no virus was detected in the brains of the older ducks, which indicating that DTMUV could more easily pass through the blood–brain barrier in younger ducks. Moreover, the highest virus titers were observed in spleens from all three experimental groups at 1 dpi. The virus titers in the lung from the youngest ducks were very significantly higher than those of 3- or 7-weeks-old infected ducks (*P* < 0.01, **Figure 3A**). At 3 dpi, maximum virus titers were achieved in the investigated tissues, except the spleen, and the virus could also be detected in the brains of 3- and 7-weeks-old ducks at this time point (**Figure 3B**). Comparing between groups, the virus titers obtained from the youngest ducks were highest, and virus titers in heart and lung were 10^1.5^–10^2.3^ higher than those of the other two groups, with highly significant difference (*P* < 0.01, **Figure 3B**). In addition, virus titers in the brains from youngest ducks were 10^1.5^ times more than in the other two groups, which was statistically significant (*P* < 0.01, **Figure 3B**). The viral titers of almost tissues began to decrease at 5 dpi, and it was noteworthy that DTMUV was not detectable in the liver and lung of 7-weeks-old infected ducks (**Figure 3C**). However, the virus titers of heart, kidney, and brain in 1-week-old ducks were at high levels, and there was a statistically significant compared with the other two groups (*P* < 0.01, **Figure 3C**). Virus titers in the ducks succumbing to infection were higher than that of the ducks euthanised on the designated days (data not shown). At 7 dpi, virus titers of all the infected ducks continually declined, and it was undetectable in the heart, liver, lung, and brain of 7-weeks-old ducks as well as the liver and lung of 3-weeks-old ducks (**Figure 3D**). At 9 dpi, all tissues from 1-weeks-old ducks showed detectable virus, but the virus titers of various tissues were lower than that obtained at 3 dpi in this age group, and titers were mostly maintained at 10^2^–10^3^ copies (**Figure 3E**). Virus titers were undetectable in the liver, spleen, lung, and brain of 3-weeks-old ducks, and similarly, no virus was found in 7-weeks-old ducks (**Figure 3E**). Generally speaking, virus titers obtained from the youngest age group of ducks were higher than those of the older during the tested time.

### Detection of IFN-γ and IL-2 in Serum

**FIGURE 4:**
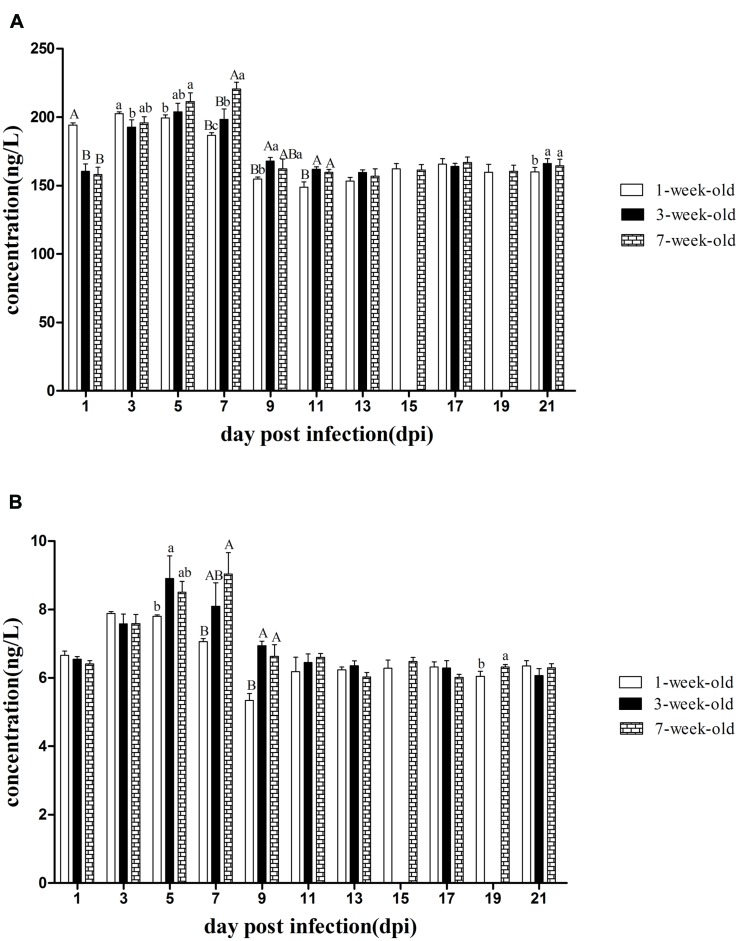
**The kinetics of IFN-γ and IL-2 in serum of different age ducks infected with DTMUV. (A)** IFN-γ levels of serum from all the infected ducks. **(B)** IL-2 levels of serum from all the infected ducks. The samples of 3-weeks-old ducks were not collected at 15 and 19 dpi. Data are expressed as mean ± SD (*n* = 3). Each sample was analyzed in triplicate. In each panel, different capital letters mean highly significant difference (*P* < 0.01), and different small letters mean significant difference (*P* < 0.05), while no letters mean no significant difference (*P* > 0.05). Significant differences were calculated by ANOVA with Duncan’s multiple range test.

As shown in **Figure 4A**, IFN-γ levels increased rapidly in 1-week-old ducks at 1 dpi, and had a highly statistically significant difference compared with that of other two groups (*P* < 0.01). At 3 dpi, although IFN-γ levels of the two older groups increased rapidly, the levels of IFN-γ continued to rise and reached a peak value in the youngest group, and there was a significant difference between 1- and 3-weeks-old ducks (*P* < 0.05). However, IFN-γ levels of 1-week-old ducks began to decline at 5 dpi, while that of 3-weeks-old ducks reached the maximum, and that of the oldest group continued to increase and had a significant difference compared with the youngest group (*P* < 0.05). At 7 dpi, the expression of IFN-γ reached the peak in the 7-weeks-old ducks, which resulted in a highly significant difference between the youngest and oldest groups (*P* < 0.01). So did the levels of IFN-γ at 9 and 11 dpi (*P* < 0.05, *P* < 0.01, respectively). Subsequently, the levels of IFN-γ in all groups were similar. However, there was a significant difference between the older groups and 1-week-old ducks at 21 dpi (*P* < 0.05).

As shown in **Figure 4B**, IL-2 levels of the three age groups were increased at 3 dpi and that of 1-week-old ducks reached a peak but there was no statistically significant difference. Consistent with the IFN-γ data, IL-2 levels of 1-week-old ducks decreased at 5 dpi, but that of the older age groups continued to increase and significant difference was exist between 1 and 3-weeks-old ducks (*P* < 0.05). At 7 dpi, IL-2 in the oldest ducks reached its maximum and had a very significantly difference compared with that of 1-week-old ducks (*P* < 0.01). Like the levels of IFN-γ, IL-2 levels also had a very significant difference among all groups at 9 dpi (*P* < 0.01). Of note was the significant difference at 19 dpi between the oldest and youngest groups (*P* < 0.05).

### Detection of Neutralizing Antibody in Serum

**FIGURE 5:**
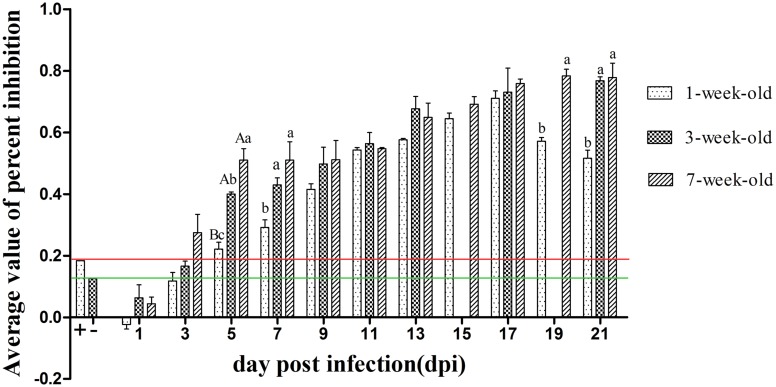
**The kinetics of serum neutralizing antibody of different age ducks infected with DTMUV**. The red and green lines represent critical threshold values of positive and negative antibody, respectively. Data are expressed as mean ± SD (*n* = 3). Each sample was analyzed in triplicate. The samples of 3-weeks-old ducks were not collected at 15 and 19 dpi. Different capital letters mean highly significant difference (*P* < 0.01), and different small letters mean significant difference (*P* < 0.05), while no letters mean no significant difference (*P* > 0.05). Significant differences were calculated by ANOVA with Duncan’s multiple range test.

As can be seen from **Figure 5**, positive serum could be detected in 7-weeks-old ducks as early as 3 dpi. A small amount of neutralizing antibody could be observed in 1-week-old ducks at 5 dpi, but the antibody titers of both older age groups had developed to high levels and were significantly higher than that of the youngest ducks (*P* < 0.05). Although the antibody titers of younger ducks continued to rise until reaching a peak value at 17 dpi, those of 3- and 7-weeks-old ducks remained at a high level at 19 and 21 dpi, and more important was that there was a significant difference between the youngest and oldest group (*P* < 0.05). Control ducks showed no seroconversion, as determined by blocking ELISA, throughout the experiment.

## Discussion

Age-related outcomes of infection in ducks have been observed with West Nile virus, Japanese encephalitis virus, Hepatitis A/B virus and highly pathogenic avian influenza virus (Jilbert et al., 1998; Pantin-Jackwood et al., 2007; Shirafuji et al., 2009; Cleton et al., 2014; Song et al., 2014). As expected, this study revealed that 1-week-old ducks infected with DTMUV demonstrated overt clinical signs of disease and high mortality (18%). The youngest infected ducks began to showed clinical symptoms as early as 2 dpi, continued to 7 dpi, and surviving ducks gradually recovered at 9 dpi. This study indicated that the latent period of serologically DTMUV-negative 1-week-old ducks was short, only about 2 days, the period of disease was contained within days 4–5, and the whole course of disease was about 7 days. The older infected ducks showed only mild symptoms and did not succumb to infection. The results of our research were similar with that of other reports (Yun et al., 2012; Sun et al., 2014), which indicted more than 3-weeks-old ducks have a strong resistance to the disease.

The gross anatomical analysis of 1-week-old ducks infected with DTMUV, especially those dying from infection, showed significant symptoms, characterized by endocardium hemorrhage, hepatomegaly, and splenomegaly, swelling of the lymphoid follicles of the ileum and meningeal hyperemia. The results of necropsy in 3-weeks-old ducks were mild, mainly restricted to endocardium bleeding, hepatomegaly, splenomegaly, and meningeal hyperemia. Furthermore, there were no significant lesions in 7-weeks-old ducks. West Nile virus could cause obvious neurological signs in host (Chancey et al., 2015). In the current study, neurological symptoms of the youngest infected ducks were observed, histological analysis revealed viral encephalitis was observed in all three groups with different degree and it was the most characteristic lesion. In addition, DTMUV could be detected in brains of the differently aged ducks (**Figure 3**). These results suggested that DTMUV had a special affinity for the central nervous system, and could cross the blood–brain barrier (Sun et al., 2014). Furthermore, degeneration, hemorrhage, necrosis, and inflammatory cell infiltration were observed in parenchymatous organs but the severity of lesions decreased as the inoculated ducks matured. The haemorrhagic inflammation of the heart was a common lesion, which may be the main reason for the onset of acute death. The observed heterophilic granulocyte inflitration in the liver and spleen of infected ducks may be related to secondary bacterial infections. In fact, a significant reduction of lymphocytes was observed in the spleen (**Figure 2K**), which would result in immunosuppression and facilitate bacterial infections. Moreover, mucosal swelling of the lymphoid follicles of the ileum and the lymphocyte necrosis suggested DTMUV might also cause damage to the gastrointestinal tissues and mucosal immunity.

The pathogenicity of virus in ducks correlated with the viral titers in tissues. Virus was detectable in all tissues except brains of 3- and 7-weeks-old ducks at 1 dpi, indicating that DTMUV could rapidly spread after it entered the body and it was a pantropic virus. Amongst the investigated tissues, virus titers in spleens from all three age groups were highest at 1 dpi, suggesting the spleen might be a target organ of DTMUV, consistent with its microscopic lesion and the findings of other researchers (Yan et al., 2011; Jiang et al., 2012; Sun et al., 2014). However, we could not exclude the possibility that this phenomenon was related to the functions of phagocytes, as virus titers within the spleen began to decrease from 3 dpi. Virus titers of heart and lung were high, which matched the endocardium hemorrhage and pneumonia occurring from 3 to 5 dpi. Virus loads of multiple tissues from 3- and 7-weeks-old ducks were at highest level at 3 dpi, but the peak was very significantly lower than that of the youngest ducks (*P* < 0.01, **Figure 3B**), which indicates that DTMUV could replicate more rapidly in 1-week-old ducks. At 5 dpi, virus titers in tissues from inoculated ducks declined quickly, particularly the 7-weeks-old ducks, virus became undetectable in the liver and lung, this phenomenon was more obvious in 3- and 7-weeks-old ducks from 7 to 9 dpi, this result showed that the older ducks could clear the virus more quickly.

Taken together, differently aged ducks had a significantly different susceptibility to DTMUV based on the clinical symptoms, necropsy analysis, histological changes and virus titers in tissues, and resistance increased as ducks matured. Here, we detected the levels of IFN-γ, IL-2, and neutralizing antibody in serum. The levels of IFN-γ and IL-2 rapidly increased but were maintained only for a short time, and highly significant difference was exist between the 1- and 7-weeks-old ducks at 7 dpi (*P* < 0.01, **Figure 4**), which showed the immune response to DTMUV in 1-week-old ducks was lower than that of the older ducks. In addition, the levels of IL-2 and IFN-γ of the youngest ducks were significant lower than those of the older ducks at 19 and 21 dpi, respectively (*P* < 0.05, **Figure 4**), which indicate 1-week-old ducks had shorter durations of immune response to virus. Neutralizing antibodies have been shown to contribute to the clearance of the virus (Kreil et al., 1998). The pattern of neutralizing antibodies of the three groups was similar to that of IFN-γ or IL-2. Although positive serum of 1-week-old ducks could be detected at 5 dpi, 3- and 7-weeks-old ducks had higher levels, and there was a highly significant difference between 1-week-old ducks and the other two groups (*P* < 0.01, **Figure 5**), and significant difference was observed between 3- and 7-weeks-old ducks (*P* < 0.05, **Figure 5**). The phenomenon may result from the destruction of the immune organs and (or) the immaturity of the immune system. Although the antibody titers of all three groups were similar from 9 to 17 dpi, reduced clearance of virus were observed in 1-week-old ducks comparing with the other two groups (**Figure 3**), which suggesting that immune system maturity was more important than the presence of antibody, despite the fact that neutralizing antibodies of 7-weeks-old ducks only existed for 3 days (**Figure 5**), but virus titers of multiple tissues were not detected at 5 dpi (**Figure 3D**). In contrast, it recently reported that the titers of neutralizing antibodies of 5-weeks-old and 5-days-old infected ducks were detected at 7 and 10 dpi, respectively (Sun et al., 2014). The reason for the difference might have the following: (a) the age of infected ducks was different, and the older ducks had a stronger immune response; (b) the immune response induced by different viral isolates was different; (c) the detection methods were different, the blocking ELISA used in this study was more sensitive than conventional neutralization test (Li et al., 2012). However, others reported that 5-weeks-old ducks inoculated DTMVU were seroconverted at 3 dpi by using the blocking ELISA method (Li et al., 2014). Due to small amount of previous research, it was necessary to further study the immune response of ducks infected with DTMUV. Moreover, the antibodies’ level of 1-week-old ducks began to decline at 19 dpi, and there was significant difference between the youngest ducks and the older ducks at 19 and 21 dpi (*P* < 0.05, **Figure 5**). The kinetics of neutralizing antibodies in infected ducks were consistent with that of IFN-γ or IL-2, which indicted that immune response to DTMUV in 1-week-old ducks was weaker than the older ducks, and this may be caused by immaturity of the immune system in 1-week-old ducks. On the whole, this study revealed the healthy 1-week-old ducks were more susceptible to DTMUV than the older ducks, and further demonstrated that the age-related immune response played a key role in resistance to DTMUV.

## Conflict of Interest Statement

The authors declare that the research was conducted in the absence of any commercial or financial relationships that could be construed as a potential conflict of interest.
